# Regulation of mitochondrial iron homeostasis by sideroflexin 2

**DOI:** 10.1007/s12576-018-0652-2

**Published:** 2018-12-20

**Authors:** Ei Ei Mon, Fan-Yan Wei, Raja Norazireen Raja Ahmad, Takahiro Yamamoto, Toshiro Moroishi, Kazuhito Tomizawa

**Affiliations:** 1 Department of Molecular Physiology, Faculty of Life Sciences, Kumamoto University, Honjo 1-1-1, Chuo-Ku, Kumamoto, 860-8556 Japan; 2 Department of Molecular Enzymology, Faculty of Life Sciences, Kumamoto University, Kumamoto, 860-8556 Japan; 3 Center for Metabolic Regulation of Healthy Aging, Faculty of Life Sciences, Kumamoto University, Kumamoto, 860-8556 Japan; 4 Precursory Research for Embryonic Science and Technology (PRESTO), Japan Science and Technology Agency (JST), Kawaguchi, 332-0012 Japan; 5 Neutron Therapy Research Center, Okayama University, Okayama, 700-8558 Japan

**Keywords:** Mitochondria, Iron, Respiration, OXPHOS, Heme

## Abstract

Mitochondrial iron is indispensable for heme biosynthesis and iron–sulfur cluster assembly. Several mitochondrial transmembrane proteins have been implicated to function in the biosynthesis of heme and iron–sulfur clusters by transporting reaction intermediates. However, several mitochondrial proteins related to iron metabolism remain uncharacterized. Here, we show that human sideroflexin 2 (SFXN2), a member of the SFXN protein family, is involved in mitochondrial iron metabolism. SFXN2 is an evolutionarily conserved protein that localized to mitochondria via its transmembrane domain. *SFXN2*-knockout (KO) cells had an increased mitochondrial iron content, which was associated with decreases in the heme content and heme-dependent enzyme activities. By contrast, the activities of iron–sulfur cluster-dependent enzymes were unchanged in *SFXN2*-KO cells. Moreover, abnormal iron metabolism impaired mitochondrial respiration in *SFXN2*-KO cells and accelerated iron-mediated death of these cells. Our findings demonstrate that SFXN2 functions in mitochondrial iron metabolism by regulating heme biosynthesis.

**Electronic supplementary material:**

The online version of this article (10.1007/s12576-018-0652-2) contains supplementary material, which is available to authorized users.

## Introduction

Iron is an essential element that is involved in the regulation of diverse biological processes, including oxygen transport, metabolism, respiration, and the cell cycle [1–3]. A large portion of cellular iron is bound to heme, which is an essential component of hemoglobin and respiratory complexes [4]. In addition, iron is utilized for the biogenesis of iron–sulfur clusters, which are indispensable for the activities of several enzymes related to RNA modification and redox signaling [5–7]. Given the essential role of iron, its deficiency often induces anemia in women and children, which can lead to severe complications such as inflammation and heart failure [8–11]. On the other hand, excess iron exerts cytotoxic effects because ferrous iron is highly active and can generate reactive oxygen species [12]. Indeed, iron overload has been linked to several diseases, such as osteoporosis, cancer, and neurological disorders [13–17].

Extracellular iron is mainly transported to the cytosol via transferrin receptor-mediated endocytosis [18]. Subsequently, a portion of cytosolic iron is transported to mitochondria for the biosynthesis of heme and iron–sulfur clusters, which are exported back to the cytosol [18]. Biosynthesis of heme and iron–sulfur clusters involves multiple chemical reactions and requires the transport of reaction intermediates across the inner and outer mitochondrial membranes [18]. Perturbation of this trafficking not only impairs the biosynthesis of heme and iron–sulfur clusters but also causes mitochondrial iron overload, leading to iron-mediated cytotoxicity.

Several mitochondrial transmembrane proteins have been implicated in the transport of iron–sulfur clusters and heme. For example, ATP-binding cassette subfamily B member 7 (ABCB7) is a transmembrane protein located in the inner mitochondrial membrane and is proposed to export iron–sulfur clusters [19, 20]. The absence of ABCB7 impairs the maturation of cytosolic iron–sulfur proteins and induces iron accumulation in mitochondria [20]. Importantly, patients carrying a defective *ABCB7* gene exhibit sideroblastic anemia, which is characterized by abnormal iron accumulation in mitochondria [21–23]. Other mitochondrial transmembrane proteins, including SLC25A38 [24], ATP-binding cassette subfamily B member 6 (ABCB6) [25], and ATP-binding cassette subfamily B member 10 (ABCB10) [26], have been implicated in the transport of reaction intermediates in heme biosynthesis. Deficiencies in these transporters result in the accumulation of intermediates and impair heme synthesis, leading to abnormal iron accumulation in mitochondria [27, 28]. However, the molecular mechanism underlying the trafficking of heme and its intermediates are unclear.

The *sideroflexin 1* (*Sfxn1*) gene was originally identified in a study of *flexed*-*tail* (*f/f*) mice, which exhibit hematological phenotypes [28, 29]. In 2001, Fleming et al. found a single base insertion in exon 2 of *Sfxn1*, which is predicted to induce a frameshift and thus lead to loss-of-function of this gene in *f/f* mice [29]. Importantly, *f/f* mice exhibit excess iron accumulation in mitochondria of erythrocytes [30, 31], suggesting that Sfxn1 is involved in mitochondrial iron homeostasis. Sfxn1 is a mitochondrial protein belonging to the SFXN protein family in mammals [29]. The mammalian *Sfxn* family consists of five members: *Sfxn1*–*Sfxn5*. The expression patterns of *Sfxn* genes differ in mouse tissues. Specifically, *Sfxn1* and *Sfxn2* are highly expressed in the liver and kidney, *Sfxn3* is ubiquitously expressed except in the lung, and *Sfxn4* and *Sfxn5* are lowly expressed in major tissues. Although the molecular functions of Sfxn proteins are unclear, recent studies demonstrated that these proteins are involved in physiological functions and disease. For example, Sfxn3 has been implicated in the differentiation of pancreatic islets in mice and the regulation of synaptic morphology at neuromuscular junctions in *Drosophila* [32, 33]. Pathogenic mutations of the *SFXN4* gene have been identified in mitochondrial disease patients with macrocytic anemia [34]. Furthermore, *SFXN4* is a susceptibility gene for common familial colorectal cancer [35].

The present study investigated the physiological role of human SFXN2, an uncharacterized SFXN family protein. We report that SFXN2 is an outer mitochondrial membrane protein that functions in mitochondrial iron homeostasis by regulating heme biosynthesis.

## Results

### SFXN2 is an evolutionarily conserved transmembrane protein

**Fig. 1:**
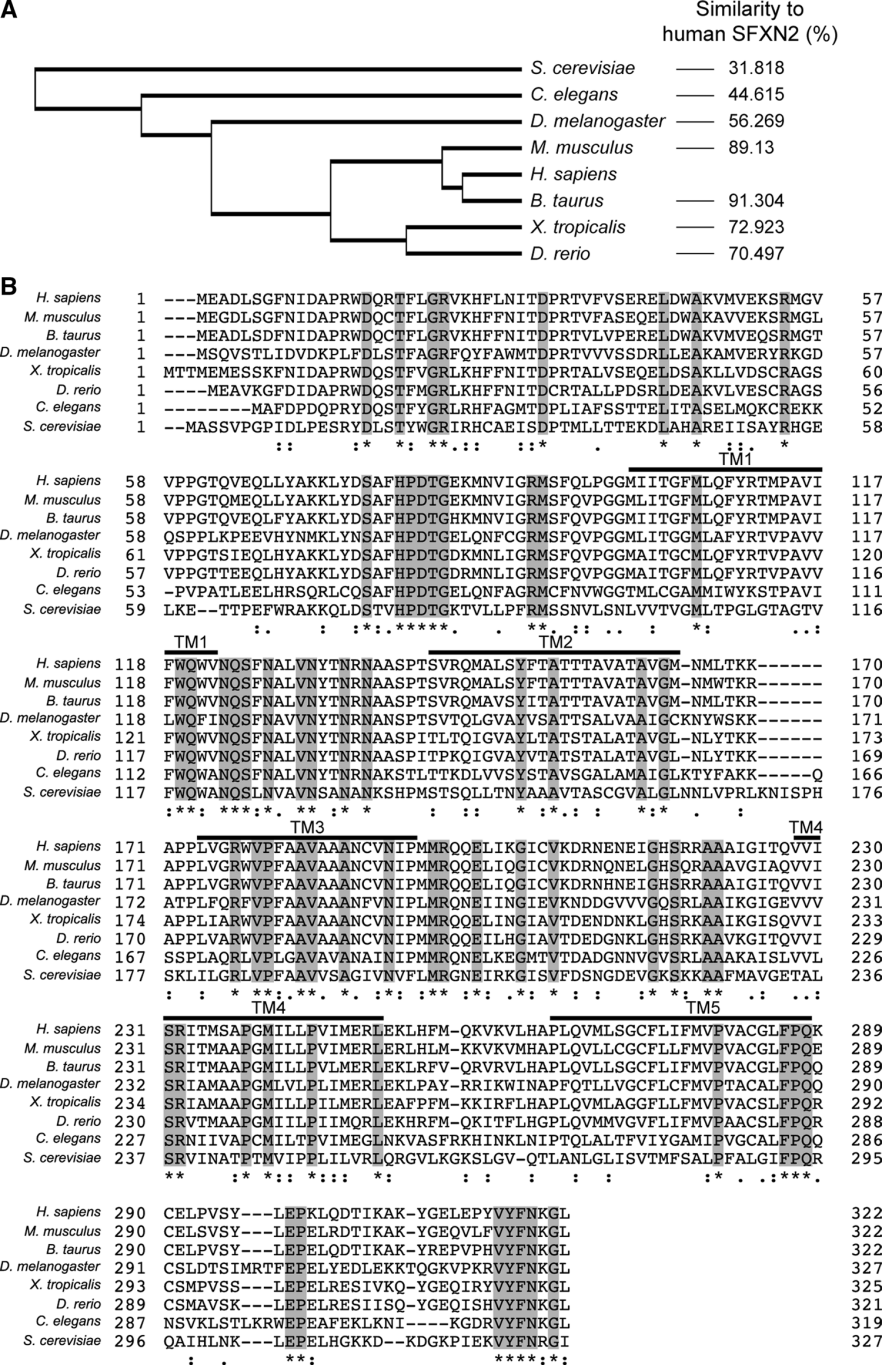
Phylogenetic analysis of SFXN2 protein. **a** Phylogenetic analysis was performed using the UniProt website. The cladogram shows the evolutionary distances between SFXN2 proteins from representative species.* Numbers on the right* show the similarity of each SFXN2 protein to human SFXN2. **b** Amino acid sequences of SFXN2 proteins were aligned using ClustalW on the UniProt website. Completely conserved residues are shaded in* gray* and labeled with* asterisks*. Residues with high and moderate homology are indicated by* colons* and* periods*, respectively. Transmembrane (TM) segments are indicated by* lines*

To examine the conservation of SFXN2 across species, we compared its sequences between eight representative eukaryotic species: *Homo sapiens*, *Mus musculus*, *Bos taurus*, *Xenopus tropicalis*, *Drosophila melanogaster*, *Caenorhabditis elegans*, *Danio rerio*, and *Saccharomyces cerevisiae*. Phylogenetic analysis demonstrated that SFXN2 is evolutionarily conversed from yeast to humans (Fig. 1a). SFXN2 proteins in vertebrates and invertebrates exhibit 70–91% and 44–56% similarity with human SFXN2, respectively. A previous in silico study demonstrated that fungal sideroflexin-1 (FSF1) is a fungal homolog of human SFXN proteins [36]. Indeed, human SFXN2 shows 31.8% similarity to yeast FSF1. Alignments of human SFXN2 with its homologs showed that conserved amino acids are evenly distributed from the N-terminus to the C-terminus. All these SFXN2 proteins contain five putative transmembrane domains and a long N-terminal region consisting of ~ 100 amino acids (Fig. 1b). The C-terminus of these SFXN2 proteins is relatively short (~ 40 amino acids), and the final eight amino acids are almost identical across the eight species.

### SFXN2 is a mitochondrial protein

**Fig. 2:**
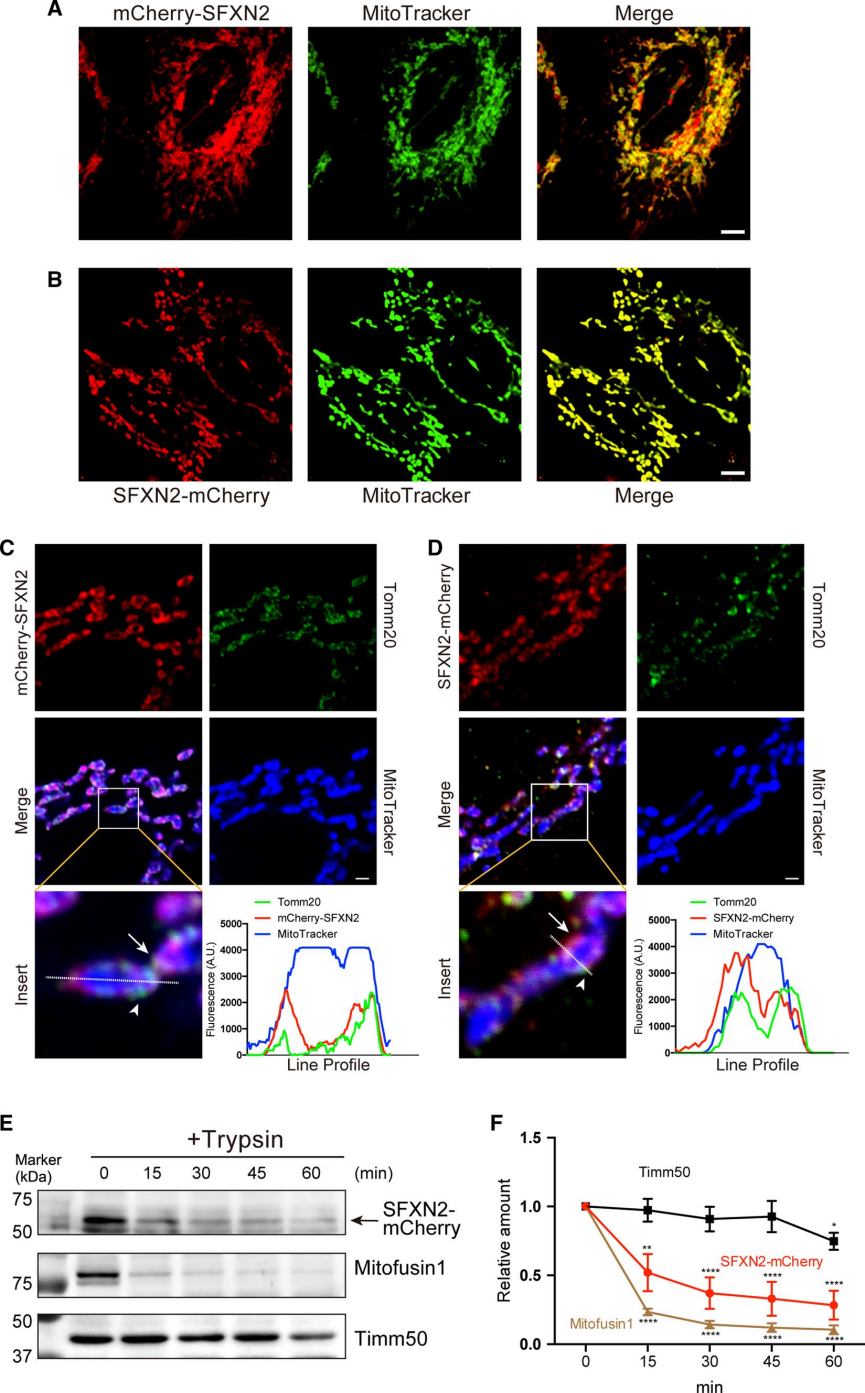
Mitochondrial localization of SFXN2. mCherry-SFXN2 (**a**) and SFXN2-mCherry (**b**) colocalized with MitoTracker.* Bar* = 5 μm. mCherry-SFXN2 (**c**) and SFXN2-mCherry (**d**) colocalized with MitoTracker and endogenous Tomm20.* Arrows* indicate mCherry-SFXN2 or SFXN2-mCherry, while* arrowheads* indicate Tomm20. The fluorescence intensities along the* dashed lines* are shown as line profile graphs.* Bars* = 1 μm. **e** Mitochondria were isolated from HEK293 cells transfected with SFXN2-mCherry and then digested with trypsin. SFXN2, Mitofusin1, and Timm50 were detected by Western blotting. The* arrow* indicates bands corresponding to SFXN2-mCherry. **f** Quantification of SFXN2-mCherry, Mitofusin1, and Timm50. *n* = 3–4 each. *p < 0.05, **p < 0.01, ****p < 0.0001 by a two-way ANOVA

We conjugated human SFXN2 to the C-terminus or N-terminus of mCherry and examined its localization in HeLa cells to verify whether it is a mitochondrial protein. Both mCherry-SFXN2 and SFXN2-mCherry colocalized with the mitochondria-specific fluorescent dye MitoTracker (Fig. 2a, b). The majority of mCherry-SFXN2 and SFXN2-mCherry colocalized with endogenous Tomm20, an outer mitochondrial membrane protein (Fig. 2c, d). Notably, some mitochondria were surrounded by mCherry-SFXN2 or SFXN2-mCherry, while the interior of mitochondria lacked mCherry fluorescent signals (Fig. 2c, d). This observation led us to speculate that SFXN2-mCherry and mCherry-SFXN2 localizes to the outer mitochondrial membrane. We purified mitochondria from HEK293 cells expressing SFXN2-mCherry and treated them with trypsin in the absence of detergent. Trypsin rapidly digested mitofusin1, which is an outer mitochondrial membrane protein, and SFXN2-mCherry (Fig. 2e, f). By contrast, Timm50, which is an inner mitochondrial membrane protein, was resistant to trypsin digestion (Fig. 2e, f). These results suggest that SFXN2-mCherry is targeted to the outer mitochondrial membrane.

### Mitochondrial targeting signal in SFXN2

**Fig. 3:**
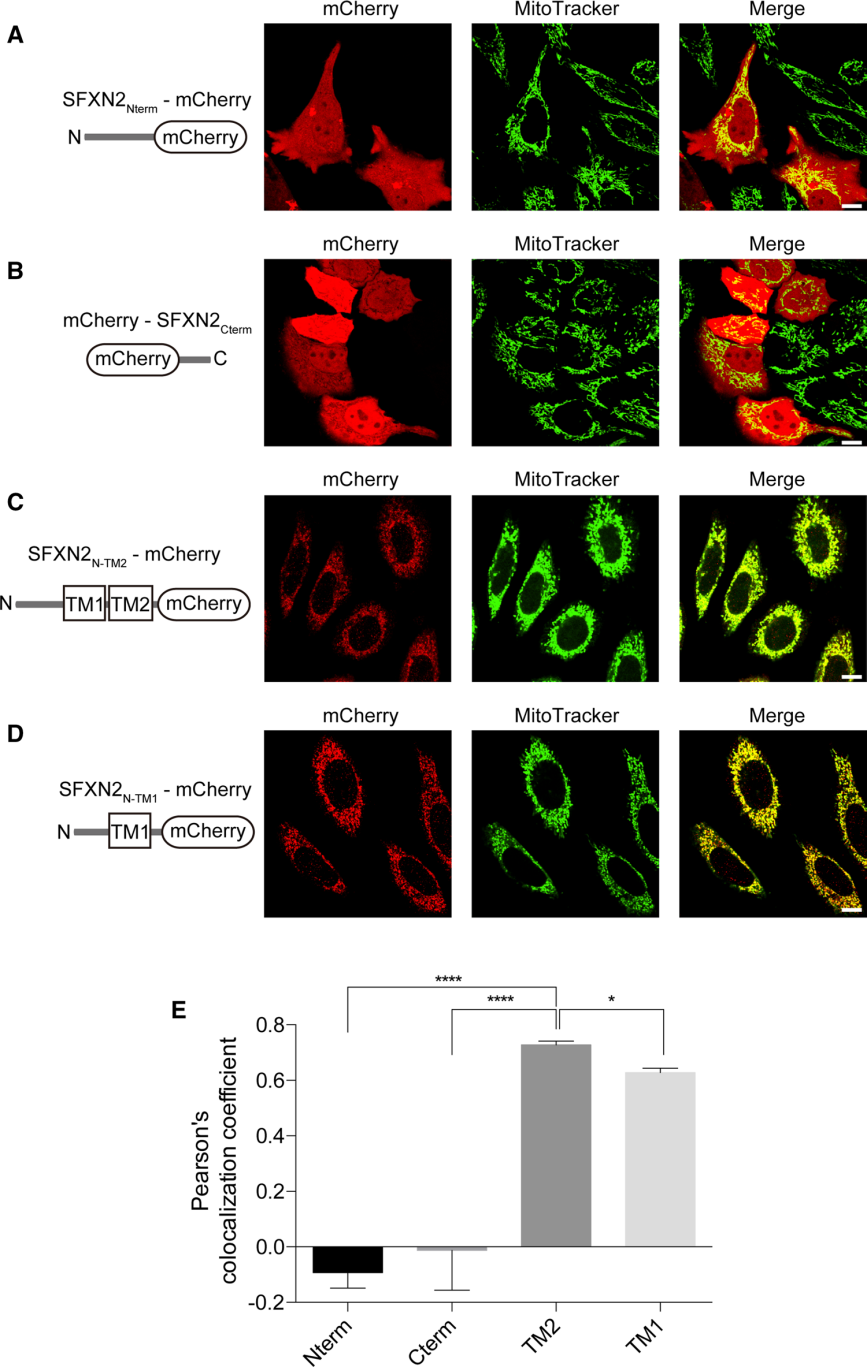
Mitochondrial targeting signal in SFXN2. The N-terminus (**a**) or C-terminus (**b**) of SFXN2 was conjugated to mCherry and then expressed in HeLa cells. SFXN2_Nterm_-mCherry and mCherry-SFXN2_Cterm_ were diffusely distributed. **c** SFXN2 was truncated at the end of the second transmembrane domain and conjugated to mCherry. The fusion protein colocalized with MitoTracker. **d** A SFXN2 fragment containing the N-terminus and the first transmembrane domain was conjugated to mCherry. The fusion protein colocalized with mitochondria.* Bars* = 5 μm. **e** Pearson’s colocalization coefficient was calculated to examine the extent of colocalization between the fluorescence of MitoTracker and the fluorescence of each chimeric protein (**a–d**). *n* = 5 for SFXN2_Nterm_-mCherry (Nterm) and mCherry-SFXN2_Cterm_ (Cterm), *n* = 16 for SFXN2_N-TM2_-mCherry (TM2), and *n* = 14 for SFXN2_N-TM1_-mCherry (TM1). *p < 0.05, ****p < 0.0001 by a one-way ANOVA

Targeting of proteins to mitochondria is very complicated, and the targeting signal might be embedded in various regions of the protein. A mitochondrial targeting signal was previously proposed to be located in the N-terminal region of SFXN proteins [36]; therefore, we conjugated the N-terminal region of SFXN2 to mCherry and examined its localization. In contrast with full-length SFXN2 shown in Fig. 2, the chimeric SFXN2_Nterm_-mCherry protein failed to localize to mitochondria (Fig. 3a). Likewise, mCherry conjugated to the C-terminal tail of SFXN2 failed to localize to mitochondria (Fig. 3b). These data demonstrate that the mitochondrial targeting signal is not located in the N-terminus or C-terminus of SFXN2; therefore, we speculated that it is present in the transmembrane domains. We truncated SFXN2 at the end of the second transmembrane domain and conjugated it to mCherry. This chimeric protein (SFXN2_N-TM2_-mCherry) colocalized with MitoTracker (Fig. 3c). Furthermore, the combination of the first transmembrane domain (TM1) and the N-terminus of SFXN2 (SFXN2_N-TM1_-mCherry) was sufficient for targeting to mitochondria (Fig. 3d). Mitochondrial targeting of SFXN2_N-TM1_-mCherry was less efficient than that of SFXN2_N-TM2_-mCherry (Pearson’s colocalization efficient = 0.728 for SFXN2_N-TM2_-mCherry and 0.627 for SFXN2_N-TM1_-mCherry, Fig. 3e).

### Iron accumulates in mitochondria of *SFXN2*-knockout (KO) cells

**Fig. 4:**
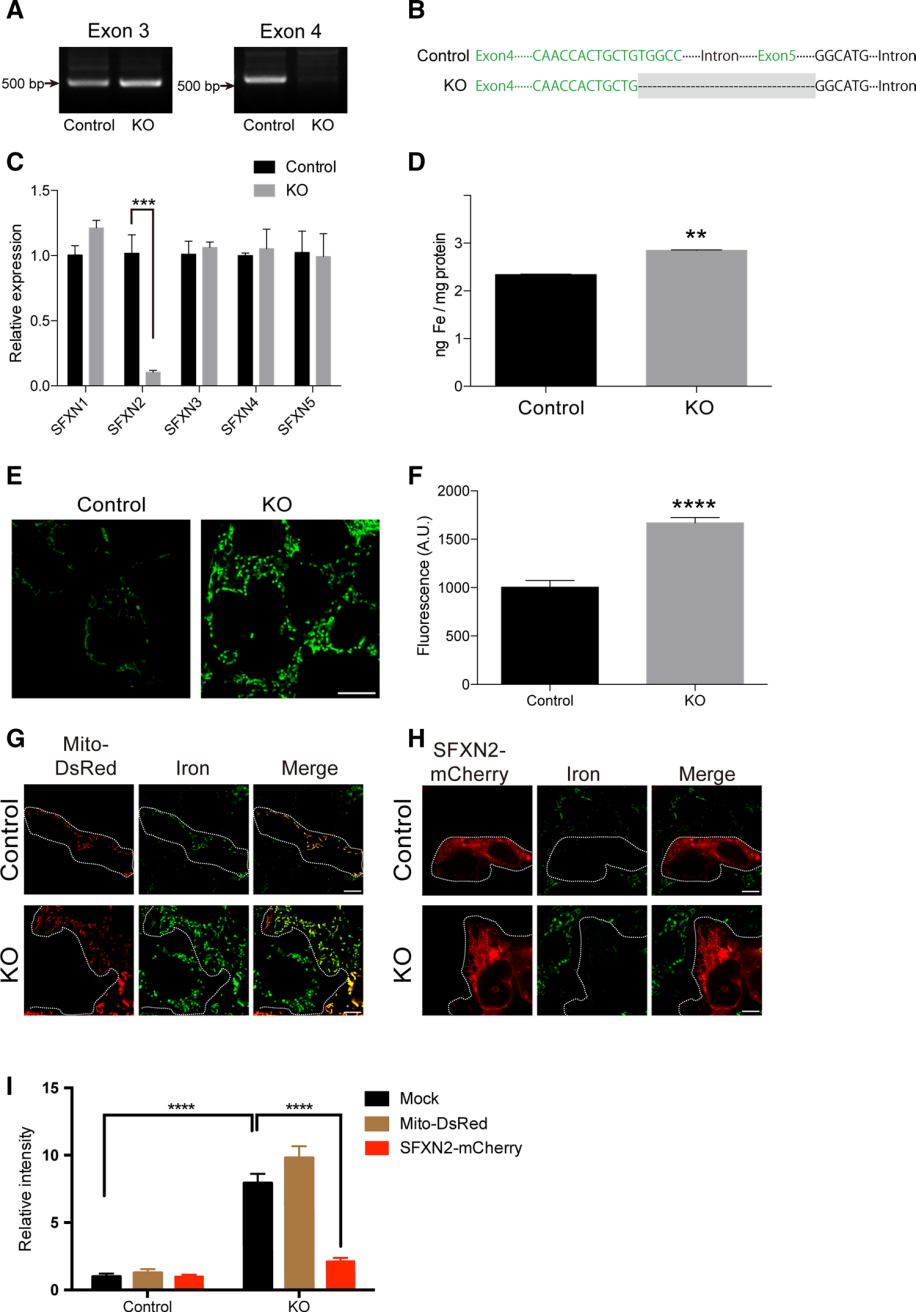
Iron accumulates in mitochondria of *SFXN2*-KO cells. **a** Genotyping of *SFXN2*-KO and control cells. **b** Alignment of DNA sequences of control and *SFXN2*-KO cells.* Green letters* show DNA regions corresponding to exon 4 and exon 5 of *SFXN2*.* Grey box* shows the deleted region in *SFXN2*-KO cells. **c** The mRNA level of *SFXN2* was significantly lower in *SFXN2*-KO cells than in control cells. Expression of *SFXN1*, *SFXN3*, *SFXN4*, and *SFXN5* did not differ between *SFXN2*-KO and control cells. *n* = 3 each. ***p < 0.001. **d** The mitochondrial iron content was measured using ICP-MS. This content was significantly higher in *SFXN2*-KO cells than in control cells. *n* = 3 each. **p < 0.01. **e** The mitochondrial iron contents of control and *SFXN2*-KO cells were investigated using Mito-FerroGreen.* Bar* = 10 μm. **f** The fluorescence intensity of Mito-FerroGreen was significantly higher in *SFXN2*-KO cells than in control cells. *n* = 100 control cells and 200 *SFXN2*-KO cells. ****p < 0.0001. **g, h** Control and *SFXN2*-KO cells were transfected with Mito-DsRed (**g**) or SFXN2-mCherry (**h**), and iron was stained with Mito-FerroGreen. **i** Quantification of the fluorescence intensity of Mito-FerroGreen in mock transfected cells and in cells transfected with Mito-DsRed and SFXN2-mCherry. *n* = 10–12. ****p < 0.0001. Expression of SFXN2-mCherry, but not of Mito-DsRed, suppressed iron accumulation in *SFXN2*-KO cells

The mitochondrial localization of SFXN2 prompted us to examine its role in mitochondrial iron homeostasis. *Sfxn2* was highly expressed in mouse kidney and liver (Fig. S1A). Notably, *SFXN2* was also expressed in human embryonic kidney 293 (HEK293) cells, and was the third most highly expressed isoform among the five *SFXN* isoforms (Fig. S1B). We then generated *SFXN2*-knockout (KO) HEK293 cells using CRISPR-Cas9-mediated genome editing [37]. We designed a guide RNA (gRNA) that targets exon 4 of *SFXN2*. After colony selection and genomic PCR, one clone showed a potential deletion near to exon 4 (Fig. 4a). Subsequent DNA sequencing revealed that a part of exon 4 and the entire exon 5 of *SFXN2* was deleted (Fig. 4b). To examine whether overall transcription of *SFXN2* was impaired by this targeted deletion, we performed quantitative PCR using primers complementary to exon 1 of *SFXN2*. The mRNA level of *SFXN2* in *SFXN2*-KO cells was 10% of that in control cells stably expressing *Cas9* (Fig. 4c). Notably, the levels of other *SFXN* family members did not differ between *SFXN2*-KO and control cells (Fig. 4c).

To examine the mitochondrial iron content, we purified mitochondria and extracted total iron using nitric acid. The extract was subjected to inductively coupled plasma-mass spectrometry (ICP-MS) for iron quantification. The iron content was significantly higher in mitochondria purified from *SFXN2*-KO cells than in mitochondria purified from control cells (Fig. 4d). ICP-MS cannot distinguish between ferric and ferrous iron when measuring the iron content; therefore, we treated control and *SFXN2*-KO cells with a fluorescent iron probe that localizes to mitochondria and specifically labels ferrous iron [38]. Confocal microscopy revealed that the fluorescence intensity of this iron probe was significantly higher in *SFXN2*-KO cells than in control cells (Fig. 4e, f). Importantly, expression of SFXN2-mCherry significantly reduced the mitochondrial ferrous iron level in *SFXN2*-KO cells, while expression of Mito-DsRed, a fluorescent mitochondrial protein, did not (Fig. 4g–i). These results clearly demonstrate that SFXN2 is required for maintenance of mitochondrial iron homeostasis.

### The heme level is decreased in *SFXN2*-KO cells

**Fig. 5:**
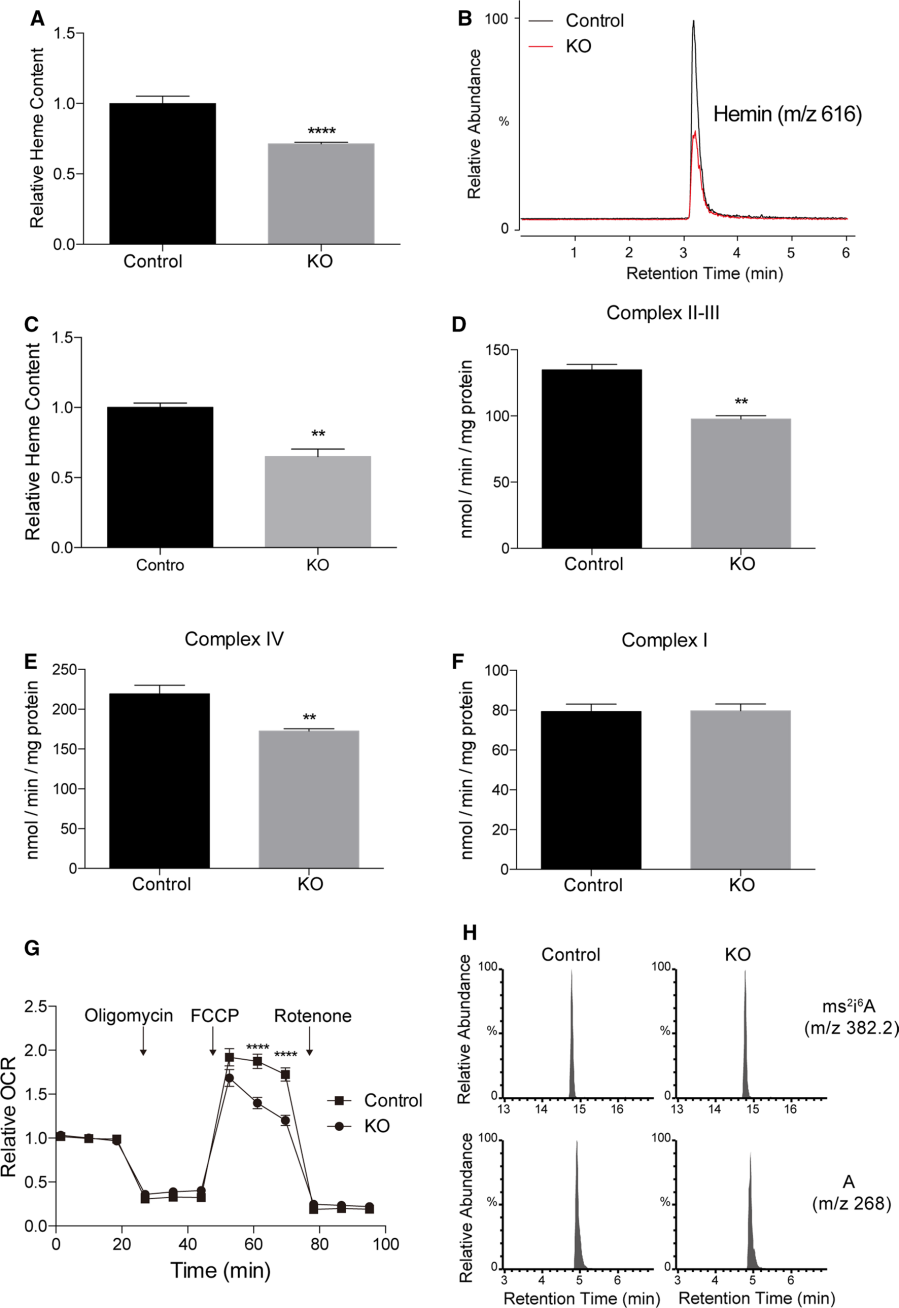
The heme level and heme-dependent enzyme activities are decreased in *SFXN2*-KO cells. **a** The relative labile heme level was lower in *SFXN2*-KO cells than in control cells. **b** Total heme (hemin) was isolated from control and *SFXN2*-KO cells. Representative mass spectra are shown. **c** The total hemin content was significantly lower in *SFXN2*-KO cells than in control cells. *n* = 3. **p < 0.01. **d, e** Activities of complexes II–III and IV were significantly lower in *SFXN2*-KO cells than in control cells. *n* = 3 each. **p < 0.01. **f** Complex I activity did not differ between *SFXN2*-KO and control cells. *n* = 3 each. **g** The relative OCR was measured in control and *SFXN2*-KO cells. The maximum OCR was significantly decreased in *SFXN2*-KO cells after application of 10 μM FCCP. *n* = 4 each. ****p < 0.0001. **h** Representative mass spectra of the ms^2^i^6^A (2-methylthio-*N*^6^-isopentenyladenosine) modification in control and *SFXN2*-KO cells. Unmodified adenosine (A) was examined as a loading control. The ms^2^i^6^A level did not differ between control and *SFXN2*-KO cells

The majority of mitochondrial iron is utilized to synthesize heme and iron–sulfur clusters [18]. Dysfunctional heme biosynthesis and iron–sulfur cluster assembly prevent efficient iron usage, leading to iron accumulation and defective respiration in mitochondria. The labile heme content is generally proportional to the total heme content [39]. Therefore, we measured the labile heme content in *SFXN2*-KO cells by monitoring peroxidase activity. The labile heme content was significantly lower in *SFXN2*-KO cells than in control cells (Fig. 5a). We also measured the total heme content by mass spectrometry. Similar to the labile heme content, the total heme content was significantly lower in *SFXN2*-KO cells than in control cells (Fig. 5b, c).

Heme and iron–sulfur clusters are essential for the activities of mitochondrial respiratory complexes. Complex I contains iron–sulfur clusters, complex IV contains heme, and complexes II and III contain both heme and iron–sulfur clusters [40]. The activities of complexes II–III and IV were significantly lower in *SFXN2*-KO cells than in control cells (Fig. 5d, e). On the other hand, the activity of complex I did not differ between *SFXN2*-KO and control cells (Fig. 5f). Collectively, the defective activities of complexes II–IV were associated with a decrease in the maximum oxygen consumption rate (OCR) in *SFXN2*-KO cells (Fig. 5g). In addition to respiratory complex activities, we examined the level of the 2-methylthio modification of mitochondrial tRNA by mass spectrometry. This modification is mediated by Cdk5 regulatory subunit-associated protein 1 (CDK5RAP1), a mitochondrial iron–sulfur cluster-dependent tRNA-modifying enzyme [41, 42]. Similar to complex I activity, the level of the 2-methylthio modification of tRNA did not differ between *SFXN2*-KO and control cells (Fig. 5h). Thus, SFXN2 might be involved in heme biosynthesis, but not in iron–sulfur cluster assembly.

**Fig. 6:**
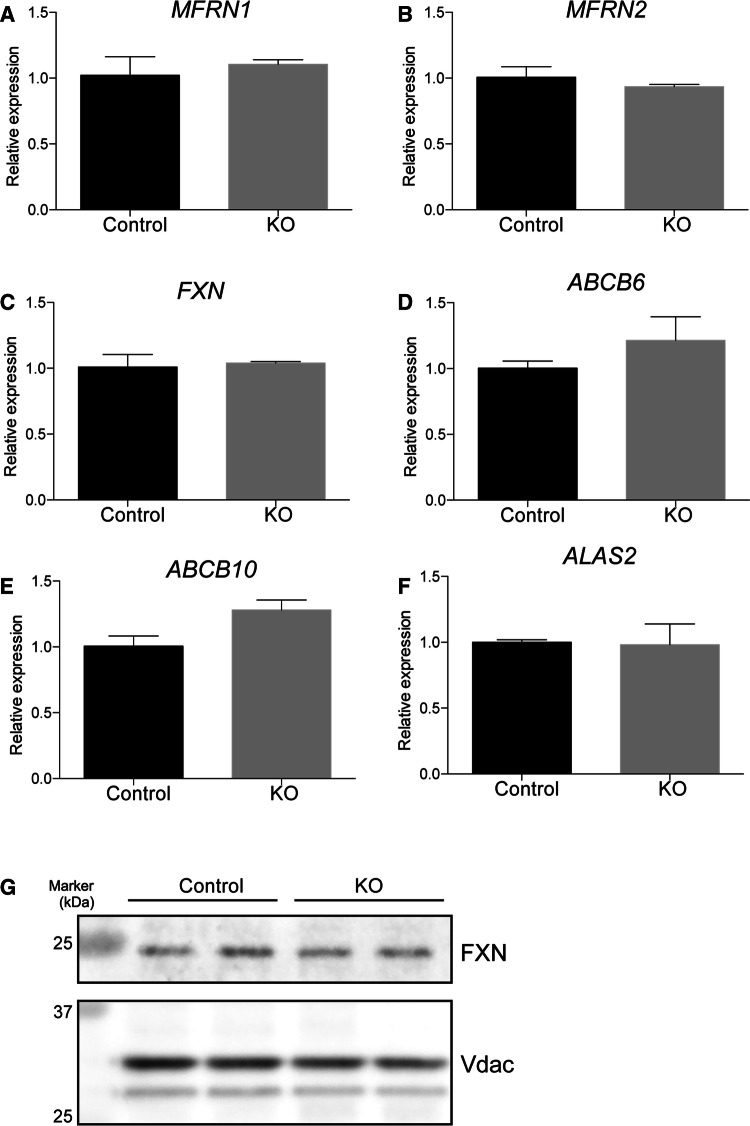
Expression of genes related to mitochondrial metabolism is unchanged in *SFXN2*-KO cells. Expression of *MFRN1* (**a**) and *MFRN2* (**b**), which are related to mitochondrial iron import, did not differ between control and *SFXN2*-KO cells. **c** Expression of *FXN*, which is related to iron–sulfur cluster assembly, did not differ between control and *SFXN2*-KO cells. Expression of *ABCB6* (**d**), *ABCB10* (**e**), and *ALAS2* (**f**), which are related to heme biosynthesis, did not differ between control and *SFXN2*-KO cells. *n* = 3 each. **g** Protein levels of FXN and Vdac in mitochondria isolated from control and *SFXN2*-KO cells were examined by Western blotting. Vdac was used as a loading control

We further examined expression of genes related to iron transport and heme biosynthesis in *SFXN2*-KO cells. Expression of *Mitoferrin1* (*MFRN1*), *Mitoferrin2* (*MFRN2*), and *Frataxin* (*FXN*), which are related to iron import and iron–sulfur cluster assembly [43, 44], did not differ between *SFXN2*-KO and control cells (Fig. 6a–c). *ABCB6*, *ABCB10*, and *ALAS2* are related to heme biosynthesis [25, 26, 45]. Expression of these genes did not differ between *SFXN2*-KO and control cells (Fig. 6d–f). In addition, we examined the protein level of FXN by Western blotting. Consistent with its mRNA expression, the protein level of FXN did not differ between *SFXN2*-KO and control cells (Fig. 6g). These results demonstrate that abnormal iron homeostasis in *SFXN2*-KO cells is not due to dysfunction of other proteins related to iron import and iron–sulfur cluster assembly.

### *SFXN2*-KO cells are sensitive to iron-mediated cytotoxicity

**Fig. 7:**
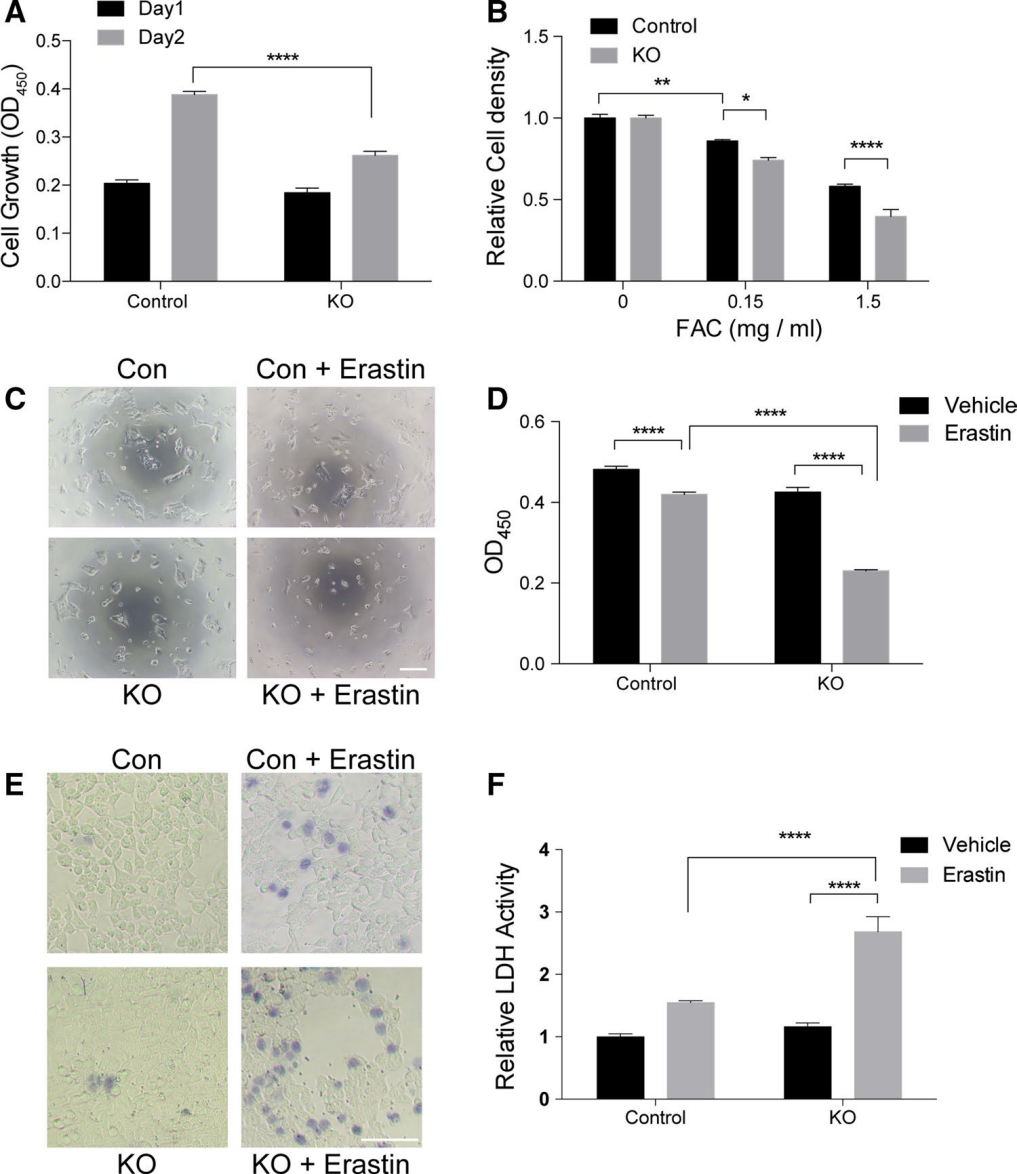
*SFXN2*-KO cells display increased sensitivity to iron. **a** Control and *SFXN2*-KO cells were seeded in 96-well plates at a density of 5000 cells/well. *SFXN2*-KO cells grew significantly slower than control cells. *n* = 7–8. ****p < 0.0001. **b** Control and *SFXN2*-KO cells were treated with the indicated concentrations of ferric ammonium citrate (FAC) for 24 h. Growth of *SFXN2*-KO cells was slowed more than that of control cells in the presence of FAC. *n* = 7–8. *p < 0.05, **p < 0.01, ****p < 0.0001. **c** Control and *SFXN2*-KO cells were treated with 10 μM erastin for 24 h and then observed under a microscope. The density of *SFXN2*-KO cells was markedly reduced upon erastin treatment.* Bar* = 200 μm. **d** Cell viability was measured using the WST-8 reagent. Erastin treatment decreased the viability of *SFXN2*-KO cells significantly more than that of control cells. *n* = 7–8. ****p < 0.0001. **e** Control and *SFXN2*-KO cells were stained with trypan blue, which labels dead cells. Erastin treatment increased the number of *SFXN2*-KO cells that were labeled with trypan blue.* Bar* = 200 μm. **f** Cell death was assessed by measuring LDH activity in the culture medium. Erastin treatment significantly increased LDH activity in the culture medium of *SFXN2*-KO cells. ****p < 0.0001

Given the decrease in mitochondrial respiration and the accumulation of iron in mitochondria, we speculated that *SFXN2*-KO cells might be sensitive to iron-induced cytotoxicity. *SFXN2*-KO cells grew significantly slower than control cells (Fig. 7a). Upon exposure to excess iron, the growth rate of *SFXN2*-KO cells was suppressed more than that of control cells (Fig. 7b). Furthermore, we treated cells with erastin in an attempt to induce cytotoxic ferroptosis, a form of iron-mediated cell death [46]. Erastin treatment significantly decreased the viability of *SFXN2*-KO cells (Fig. 7c, d). In addition, we assessed cell death by performing trypan blue staining and monitoring release of lactate dehydrogenase (LDH) into the culture medium (Fig. 7e, f). Erastin treatment increased the number of *SFXN2*-KO cells stained with trypan blue (Fig. 7e) and significantly augmented release of LDH into the culture medium by *SFXN2*-KO cells (Fig. 7f). Taken together, these results demonstrate that *SFXN2*-KO cells are sensitive to iron toxicity.

## Discussion

The present study demonstrated that human SFXN2 is a mitochondrial protein that regulates mitochondrial iron homeostasis. *SFXN2*-KO cells exhibited abnormal mitochondrial iron accumulation, which was associated with a decrease in mitochondrial respiration. In addition, *SFXN2*-KO cells displayed a growth defect upon iron overload and were susceptible to erastin-induced death. These results demonstrate that SFXN2 is important for maintenance of mitochondrial iron homeostasis. Iron homeostasis is also perturbed in *Sfxn1*- and *SFXN4*-deficient cells [29, 34]; therefore, it is conceivable that SFXN family proteins are evolutionarily conserved to regulate mitochondrial iron homeostasis.

What is the role of SFXN2 in mitochondrial iron homeostasis? Iron is imported from the cytosol into mitochondria for biosynthesis of heme and assembly of iron–sulfur clusters, which are subsequently exported back to the cytosol. Defects in genes related to heme biosynthesis or iron–sulfur cluster assembly lead to aberrant iron accumulation in mitochondria [18]. The high mitochondrial iron content in *SFXN2*-KO cells was associated with decreases in the heme content and heme-dependent enzyme activities. By contrast, the activities of iron–sulfur cluster-dependent enzymes were unchanged in *SFXN2*-KO cells. These results suggest that SFXN2 is involved in heme biosynthesis, but not in iron–sulfur cluster assembly.

Heme biosynthesis involves multiple enzymatic reactions that occur sequentially in the cytosol and mitochondria. Accordingly, the intermediates of these chemical reactions must be imported into and exported from mitochondria. Despite the complexity, only a few transmembrane proteins have been reported to mediate this transport. For example, the inner mitochondrial membrane protein SLC25A38 transports glycine, which is required for the first step of heme synthesis [24], while ABCB6 and ABCB10 are involved in import of protoporphyrin into mitochondria [28, 47]. However, these transporters cannot explain the entire heme transport system. Interestingly, a very recent study revealed that SFXN1 is a mitochondrial serine transporter [48]. Serine hydroxymethyltransferase 2 metabolizes serine into glycine in mitochondria; therefore, loss of SFXN1 reduces de novo glycine synthesis in mitochondria, leading to defects in one-carbon metabolism including purine synthesis. Given the essential role of glycine in biosynthesis of heme, it is conceivable that defective heme synthesis in *SFXN2*-KO cells is due to impaired serine transport and de novo glycine synthesis. A further study is required to elucidate the role of SFXN2 in serine transport and its relevance to heme biosynthesis.

SFXN2 (and SFXN1) is predominantly expressed in the liver and kidney, and is lowly expressed in other tissues. This differential expression pattern might be due to differences in the requirement for hemoglobin and heme synthesis. The fetal liver is the major organ involved in erythropoiesis during the late phase of embryogenesis [49]. In adults, the liver and cortex of the kidney have a high heme-synthesizing capacity, which sustains the activity of cytochrome P450 to detoxify endogenous and exogenous substances [50]. The high demand for hemoglobin and heme in the liver and kidney might explain why *SFXN2* is highly expressed in these organs. On the other hand, despite its low abundance, SFXN2 is also required for synthesis of heme and purine nucleotides to sustain cellular activity in other tissues. SFXN3–SFXN5 may compensate for low expression of SFXN2 in these tissues. A further study using knockout mice is required to elucidate the physiological roles of SFXN2 and other SFXN family members.

Proteins are targeted to mitochondria by specific signals. The targeting signals are usually located in the N-terminal, internal, and C-terminal regions of mitochondrial proteins with a single transmembrane segment [51]. Some outer mitochondrial membrane proteins, such as peripheral benzodiazepine receptor, contain several transmembrane segments, and the location of the targeting signal is unclear [52]. A previous in silico study predicted that the targeting signal of SFXN2 is located in its N-terminal region [36]. However, neither the N-terminus nor the C-terminus was sufficient for targeting of SFXN2 to mitochondria in the present study. Instead, the transmembrane domains of SFXN2 functioned as a mitochondrial targeting signal and guided SFXN2 to the mitochondria. In contrast to SFXN2, SFXN3, and SFXN4 are targeted to the inner mitochondrial membrane [33, 34]. Sequence homology is 54% between SFXN2 and SFXN3, but only 20% between SFXN2 and SFXN4 (Fig. S2). SFXN3 contains one fewer putative transmembrane domains, which are potentially important for mitochondrial targeting (Fig. 3), than SFXN2 (Fig. S2). A further study is needed to elucidate the localizations of endogenous SFXN proteins.

All *SFXN* family genes are expressed in HEK293 cells, but only *SFXN2* was deleted in the present study. Given the moderate decrease in the heme level, the remaining SFXN proteins likely compensated for the loss of SFXN2 function. Consistently, the hematological phenotypes are mild in Sfxn1-deficient *f/f* mice [29], but severe in transgenic mice with defective heme biosynthesis [28, 45]. Simultaneous KO of multiple *SFXN* genes is required to elucidate the functions of SFXN proteins.

In conclusion, SFXN2 is a mitochondrial membrane protein that regulates heme biosynthesis and contributes to mitochondrial iron homeostasis.

## Materials and methods

### Cell culture

HEK293 and HeLa cells were cultured in Dulbecco’s modified Eagle’s medium (DMEM; Invitrogen) supplemented with 4500 mg/l glucose, 10% fetal bovine serum (Corning), and 1 × penicillin/streptomycin (Invitrogen) in an incubator containing 5% CO_2_ at 37 °C.

### Plasmids and cDNA

Human SFXN2 was amplified from human cDNA derived from HEK293 cells and subcloned into the pEF1a-mCherry-C1 and pEF1a-mCherry-N1 vectors (Clontech). pDsRed2-Mito was obtained from Clontech. For the generation of lentiviruses, lentiCRISPRv2 (#52961), psPAX2 (#12260), and pVSVg (#8454) were purchased from Addgene.

### Generation of *SFXN2*-KO cells

A gRNA targeting exon 4 of *SFXN2* was selected using the CRISPR DESIGN web tool. DNA oligonucleotides were annealed and ligated into LentiCRISPRv2 that had been digested with BsmBI (New England BioLabs). Lentiviruses were generated in HEK293FT cells according to the manufacturer’s instructions. Culture media containing viruses were filtered and stored at − 80 °C until use. HEK293 cells were infected with lentiviruses carrying the *Cas9* gene and gRNA. Two days later, cells were seeded in 10-cm culture dishes at a density of 40 cells/dish in the presence of 2 μg/ml puromycin. Colonies were plated in 96-well dishes and subjected to genotyping.

### Immunocytochemistry

HeLa and HEK293 cells were transfected with mCherry-SFXN2 or SFXN2-mCherry using Lipofectamine 3000. Cells were stained with 100 nM MitoTracker Green FM (Invitrogen) or 100 nM MitoTracker Deep Red FM (Invitrogen) for 30 min. Thereafter, cells were fixed with 4% paraformaldehyde for 10 min at room temperature. Finally, cells were stained with an anti-Tomm20 antibody (Abcam, ab186734) and an anti-mCherry antibody (Abcam, ab125096), and then incubated with secondary antibodies conjugated with Alexa Fluor 488 and Alexa Fluor 546. Fluorescence was observed and analyzed using a confocal microscope (Olympus 3000) and FluoView software.

### Iron staining

To label iron in mitochondria, cells were incubated with Mito-FerroGreen (Dojindo) in accordance with the manufacturer’s instructions. Briefly, cells were washed thrice with serum-free DMEM. Mito-FerroGreen was freshly dissolved in dimethyl sulfoxide (Sigma) and added to cells at a final concentration of 5 μM. Cells were incubated with the reagent for 30 min at 37 °C and then washed with phosphate-buffered saline (PBS). Fluorescence was observed using a confocal microscope.

### Isolation of mitochondria

Cells were grown in 10-cm dishes until they reached 80% confluency, washed with PBS, and suspended in homogenization buffer (250 mM sucrose, 20 mM Mops, 1 mM EDTA, and a protease inhibitor cocktail (Sigma)). Thereafter, cells were homogenized with a Teflon homogenizer until 70–80% of cells were lysed. The homogenate was centrifuged at 800*g* for 10 min at 4 °C, and the supernatant was centrifuged at 8000*g* for 10 min. The resulting pellet was used as the crude mitochondrial fraction. Frataxin was detected using an antibody (Abcam, ab175402).

### Trypsin digestion

Isolated mitochondria were incubated with 25 μg/ml trypsin (Promega) on ice in homogenization buffer for the indicated durations. A trypsin inhibitor (Sigma) was added to stop the digestion. Mitochondria were washed with homogenization buffer and subjected to SDS-PAGE. Mitofusin1 and Timm50 were selected as representative outer and inner mitochondrial membrane proteins, respectively. Mitofusin1, Timm50, and mCherry were detected using specific antibodies (ab104274, ab109436, and ab125096, respectively) purchased from Abcam.

### Measurement of mitochondrial respiratory complex activities

The activities of mitochondrial complexes I, II–III, and IV were measured using previously described methods [41]. To measure complex I activity, 50 μg of isolated mitochondria was mixed with 50 mM potassium phosphate (pH 7.4), 2 mM KCN, 75 μM NADH (nicotinamide adenine dinucleotide reduced disodium salt), and 50 μM coenzyme Q1. Absorbance at 340 nm was measured for 200 s. To measure complex II–III activity, 50 μg of isolated mitochondria was mixed with 50 mM potassium phosphate (pH 7.4), 20 mM succinate, 0.5 mM EDTA, 2 mM KCN, and 30 μM cytochrome C. Absorbance at 550 nm was measured for 200 s. To measure complex IV activity, 50 µg of isolated mitochondria was mixed with 10 mM potassium phosphate (pH 7.4) and 10 μM ferrocytochrome C. Absorbance at 550 nm was measured for 200 s.

### Flux analysis

Oxygen consumption was measured using a Seahorse XF Analyzer (Agilent), according to the manufacturer’s instructions. Briefly, cells were seeded in a 24-well culture plate (Agilent) at a density of 50,000 cells/well. Oligomycin (Sigma), carbonyl cyanide 4-(trifluoromethoxy) phenylhydrazone (FCCP, Sigma), and rotenone (Sigma) were added sequentially and oxygen consumption was measured under each condition.

### Cell viability

Control and *SFXN2*-KO cells were seeded in a 96-well plate at a density of 5000 cells/well. The following day, cells were treated with 10 μM erastin (Sigma) and incubated for 24 h. Cell viability was monitored using WST-8 reagent (Dojindo) according to the manufacturer’s instructions. Absorbance was measured at 405 nm. To investigate cell death, control and *SFXN2*-KO cells were seeded in a 96-well plate at a density of 10,000 cells/well. The following day, cells were treated with 10 μM erastin (Sigma) and incubated for 24 h. Cell death was assessed by the LDH assay and trypan blue staining in the same well. The culture medium in each well was transferred to a fresh 96-well plate. The amount of LDH released into the culture medium was measured using a Cytotoxicity LDH Assay Kit-WST (Dojindo) according to the manufacturer’s instructions. Cells were stained with trypan blue (Sigma) and examined under a microscope (Olympus) equipped with a CCD camera.

### Quantitative PCR

Total RNA was extracted from cells using TRIzol (Invitrogen) according to the manufacturer’s instructions. cDNA was generated using PrimeScript RT Master Mix (Takara), and then quantitative PCR was performed using TB Green Premix Ex Taq (Takara) and a Rotor-Gene Q system (Qiagen). The primer sequences are provided in Supplementary Table 1. The level of the target gene was normalized against that of *18S rRNA* for analyses in mouse tissues, while the levels of *SFXN1*–*SFXN5* were normalized against that of *Ubiquitin C* (*UBC*) for analyses in HEK293 cells. Relative expression was calculated using the $2^{{\Delta \Delta {\text{C}}_{t} }}$ method as described previously [53]. Expression levels of target genes were normalized against that of 18S rRNA.

### Mass spectrometry

The total mitochondrial iron content was measured using ICP-MS (Agilent 7900). Mitochondria were suspended in 2% nitric acid and heated at 80 °C for 15 min to extract iron. Denatured proteins were pelleted by centrifugation at 10,000*g* for 15 min, and the supernatant was subjected to ICP-MS analysis according to the manufacturer’s instructions.

The level of the 2-methylthio-*N*^6^-isopentenyladenosine modification of mitochondrial tRNA was measured using liquid chromatography-mass spectrometry (Shimadzu LCMS-8050) as previously described [42]. Briefly, total RNA was purified from control and *SFXN2*-KO cells. Five micrograms of total RNA was digested with nuclease P1 (Fujifilm-Wako). Nucleosides were fractionated using an Inertsil ODS-3 column and analyzed in multiple reaction monitoring mode.

### Measurement of labile heme

After reaching 80% confluency, cells were washed with PBS, lysed in Tris-buffered saline containing 1% Triton X-100, sonicated, and centrifuged at 10,000*g* for 10 min. The heme content was measured using a Hemin Assay kit (Abcam, ab65332), which utilizes peroxidase activity to measure the level of heme. Protein concentrations were measured using a Pierce BCA assay kit (Thermo Fisher Scientific). Peroxidase activity was normalized against the total protein concentration.

### Measurement of total heme

The total heme (hemin) content was measured by mass spectrometry according to a previously described method [54]. Briefly, 2 × 10^7^ cells were suspended in 1 ml of PBS, and an aliquot of the cell suspension was transferred to a fresh tube for measurement of the total protein concentration. The cell suspension was mixed with 3 ml of acetonitrile to extract heme from proteins. Next, 2 ml of acetonitrile/HCl (8:2) was added to the sample, followed by 0.5 ml of saturated MgSO_4_ and 0.05 g of NaCl. After brief centrifugation at 2600*g* for 5 min, the upper organic phase, which contained free hemin, was analyzed by mass spectrometry (Agilent 6470). The peak corresponding to hemin was normalized against the protein concentration.

### Statistical analysis

At least three independent replicates were performed in all experiments. All data were analyzed using GraphPad Prism 6 software. The unpaired Student’s *t* test was used to assess the significance of differences between two groups. A two-way ANOVA followed by Tukey’s multiple comparison test was used to examine the significance of differences between more than two groups. A two-tailed *p* value of 0.05 was considered significant. Data are presented as the mean ± SEM.

### Electronic supplementary material

Below is the link to the electronic supplementary material.

Supplementary material 1 (PDF 541 kb)
