# Supplementary material for: Regulation of mitochondrial iron homeostasis by sideroflexin 2
Source: J Physiol Sci. 2018 Dec 20;69(2):359–73. doi: 10.1007/s12576-018-0652-2 (PMC6373408; doi:10.1007/s12576-018-0652-2)
Supplement: Supplementary file 1 — Supplementary material 1 (PDF 541 kb) [file 12576_2018_652_MOESM1_ESM.pdf]

Supporting Information

Regulation of mitochondrial iron homeostasis by sideroflexin 2

Ei Ei Mon[1], Fan-Yan Wei[1,4*], Raja Norazireen Raja Ahmad[1], Takahiro Yamamoto[1], Toshiro Moroishi[2,3,4], & Kazuhito Tomizawa[1,3,5*]

Departments of [1]Molecular Physiology and [2]Molecular Enzymology, Faculty of Life Sciences, Kumamoto University, Kumamoto 860-8556, Japan

[3]Center for Metabolic Regulation of Healthy Aging, Faculty of Life Sciences, Kumamoto University, Kumamoto 860-8556, Japan

[4]Precursory Research for Embryonic Science and Technology (PRESTO), Japan Science and Technology Agency (JST), Kawaguchi 332-0012, Japan

[5]Neutron Therapy Research Center, Okayama University, Okayama 700-8558, Japan

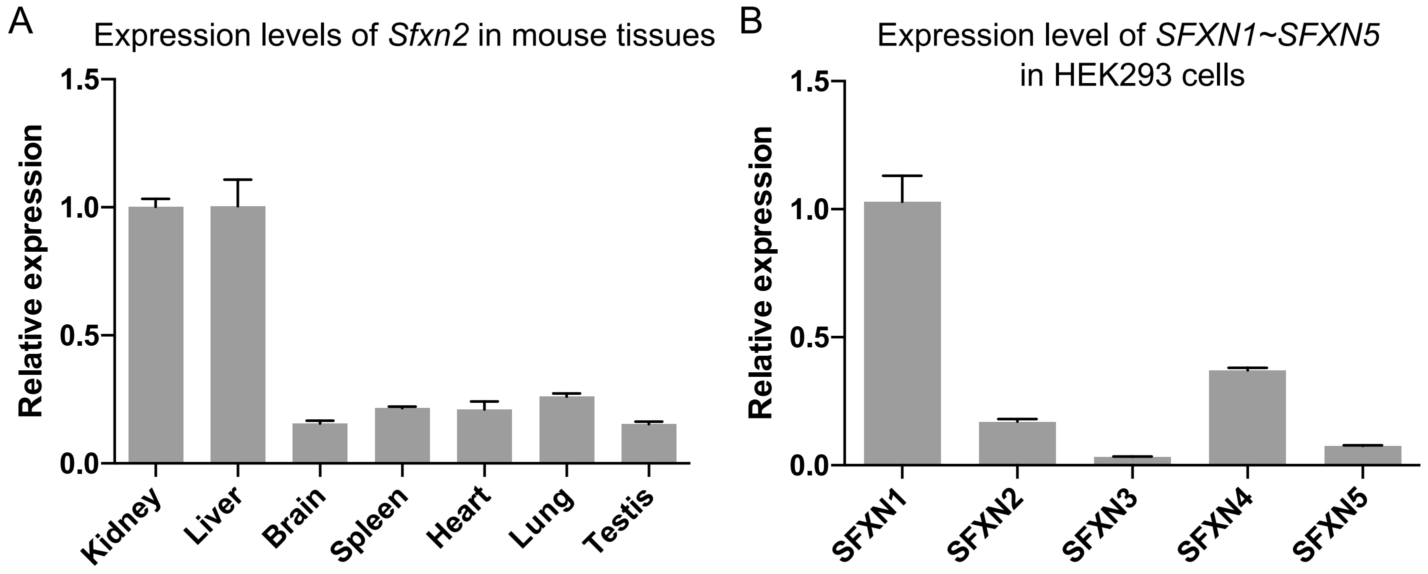

Supplementary Figure 1. Expression levels of *Sfxn2* in mouse tissues and of *SFXN1–SFXN5* in HEK293 cells. (A) Total RNA was isolated from various mouse tissues. The expression level of *Sfxn2* was examined by quantitative PCR. n = 4 per group. (B) Expression levels of *SFXN1–SFXN5* in HEK293 cells were examined by quantitative PCR. n = 4 per group.

```
SFXN1    1   ------------------MSGELPPNIN---IKEPRWDQSTFIGRANHFFTVTDPRNILL      39
SFXN2    1   --------------------MEADLSGFN---IDAPRWDQRTFLGRVKHFLNITDPRTVFV      38
SFXN3    1   --------------------MGELPLDIN---IQEPRWDQSTFLGRARHFFTVTDPRNLLL      38
SFXN4    1   MSLEQEEETQPG---RLLGRRDAVPAFIEPNVRFWITERQSFIRRFLQWTELLDPTNVFI      57
SFXN5    1   --MADTATTASAAAASAASASSDAPPFQ---LGKPRFQQTSFYGRFRHFLDIIDPRTLFV      55
                                       :    :     :: :*  *  ::  : ** .:::

SFXN1   40   TNEQLESARKIVHDYRQGIVPPGLTENELWRAKYIYDSAFHPDTGEKMILIGRMSAQVPM      99
SFXN2   39   SERELDWAKVMVEKSRMGVVPPGTQVEQLLYAKKLYDSAFHPDTGEKMNVIGRMSFQLPG      98
SFXN3   39   SGAQLEASRNIVQNYRAGVVTPGITEDQLWRAKYVYDSAFHPDTGEKVVLIGRMSAQVPM      98
SFXN4   58   SVESIENSRQLLCTNE-DVSSPASADQRIQEAWKRSLATVHPDSSNLIPKLFRPAAFLPF     116
SFXN5   56   TERRLREAVQLLEDYKHGTLRPGVTNEQLWSAQKIKQAILHPDTNEKIFMPFRMSGYIPF     115
             :   :  :  ::   . .   *.   :.:  *     : .***:.: :    * :  :*

SFXN1  100   NMTITGCMMTFYRTTPAVLFWQWINQSFNAVVNYTNRSGDAPLTVNELGTAYVSATTGAV     159
SFXN2   99   GMIITGFMLQFYRTMPAVIFWQWVNQSFNALVNYTNRNAASPTSVRQMALSYFTATTTAV     158
SFXN3   99   NMTITGCMLTFYRKTPTVVFWQWVNQSFNAIVNYSNRSGDTPITVRQLGTAYVSATTGAV     158
SFXN4  117   MAPTVFLSMTPLKGIKSVILPQVFLCAYMAAFNSIN--GNRSYTCKPLERSLLMA--GAV     172
SFXN5  116   GTPIVVGLLLPNQTLASTVFWQWLNQSHNACVNYANRNATKPSPASKFIQGYLGAVISAV     175
                 .   :   :   :.:: * .  :. * .*  *  .        :  . . *   **

SFXN1  160   ATALGLNALTKHV-------SPLIGRFVPFAAVAAANCINIPLMRQRELKVGIPVTDENG     212
SFXN2  159   ATAVGMNMLTKKA-------PPLVGRWVPFAAVAAANCVNIPMMRQQELIKGICVKDRNE     211
SFXN3  159   ATALGLKSLTKHL-------PPLVGRFVPFAAVAAANCINIPLMRQRELQVGIPVADEAG     211
SFXN4  173   ASSTFLGVIPQFVQMKYGLTGPWIKRLLPVIFLVQASGMNVYMSRSLESIKGIAVMDKEG     232
SFXN5  176   SIAVGLNVLVQKANKFTPATRLLIQRFVPFPAVASANICNVVLMRYGELEEGIDVLDSDG     235
             : :  :  : :            : * :*.  :. *.  *: : *  *   ** * *

SFXN1  213   NRLGESANAAKQAITQVVVSRILMAAPGMAIPPFIMNTLEKKAFLKRFPWMSAPIQVGLV     272
SFXN2  212   NEIGHSRRAAAIGITQVVISRITMSAPGMILLPVIMERLEKLHFMQKVKVLHAPLQVMLS     271
SFXN3  212   QRLGYSVTAAKQGIFQVVISRICMAIPAMAIPPLIMDTLEKKDFLKRRPWLGAPLQVGLV     271
SFXN4  233   NVLGHSRIAGTKAVRETLASRIVLFGTSALIPEVFTYFFKRTQYFRKNPGSLWILKLSCT     292
SFXN5  236   NLVGSSKIAARHALLETALTRVVLPMPILVLPPIVMSMLEKTALLQARPRLLLPVQSLVC     295
             : :* *  *.  .: :.  :*: :      :  ..   :::   ::        ::

SFXN1  273   GFCLVFATPLCCALFPQKSSMSVTSLEAELQAKIQESHPELR-RVYFNKGL              322
SFXN2  272   GCFLIFMVPVACGLFPQKCELPVSYLEPKLQDTIKAKYGELEPYVYFNKGL              322
SFXN3  272   GFCLVFATPLCCALFPQKSSIHISNLEPELRAQIHEQNPSVE-VVYYNKGL              321
SFXN4  293   VLAMGLMVPFSFSIFPQIGQIQYCSLEEKIQSPTEE------TEIFYHRGV              337
SFXN5  296   LAAFGLALPLAISLFPQMSEIETSQLEPEIAQATSS------RTVVYNKGL              340
                : :  *.. .:***  .:    ** ::              : :::*:
```

Supplementary Figure 2: The amino acids sequences of human SFXN1~SFXN5 were aligned. Yellow boxes indicate putative transmembrane domains.

Supplementary Table 1-Primer sequences

Genes	Sequence (5' to 3')
Human_MFRN1_f	CGGTGGACTCGGTGAAGAC
Human_MFRN1_r	GGGCTCCGTAGATACTTGTGTA
Human_MFRN2_f	GGCTGAACGTCACAGCAAC
Human_MFRN2_r	GCACCATTGGCAATATGGCT
Human_FXN_f	GGAAACGCTGGACTCTTTAGC
Human_FXN_r	CCAGTTTGACAGTTAAGACACCA
Human_ABCB6_f	TGGTGCTGATTCGCTGTCTTG
Human_ABCB6_r	CAGAAGTAGATAGCTTGGCAGTG
Human_ABCB10_f	TGGATTTCTCACGATGTCCAGT
Human_ABCB10_r	ACAGTGGGGTTGGTATAGATGAC
Human_ALAS2_f	ACCTACCGTGTGTTCAAGACT
Human_ALAS2_r	AGATGCCTCAGAGAAATGTTGG
Human_SFXN1_f	GATGACGTTTTACAGGACTACGC
Human_SFXN1_r	ATTGACGACGGCATTGAAGGA
Human_SFXN2_f	GTCAATATCCCCATGATGCGAC
Human_SFXN2_r	AGCTCTCCGGGAATGACCAA
Human_SFXN3_f	ATCGTGCAGAACTACAGGGC
Human_SFXN3_r	ATGGAAGGCGGAGTCATACAC
Human_SFXN4_f	CGCTTCTGGATCACCGAGC
Human_SFXN4_r	CTGTCGGGATGCACTGTTG
Human_SFXN5_f	CCTCGCACACTCTTTGTCACT
Human_SFXN5_r	AGGGTCCCATGCTTATAGTCC
Human_UBC_f	CTGGAAGATGGTCGTACCCTG
Human_UBC_r	GGTCTTGCCAGTGAGTGTCT
Mouse_Sfxn1_f	GTGCCACCCAACATTAACATCA
Mouse_Sfxn1_r	ACCACTTTCCTCGCATTCTCTA
Mouse_Sfxn2_f	GCGGATCTGTCTGGCTTTAAT
Mouse_Sfxn2_r	CTGCTCTGATGCAAAGACCGT
Mouse_Sfxn3_f	CAGGAAGACTCCGACTGTGG
Mouse_Sfxn3_r	CCGCTGCGATTAGAGTAATTCAC

Mouse_Sfxn4_f	TCGAAGATTTTGTCAGTGGATGG
Mouse_Sfxn4_r	AGATGGGGGATCAACTTGCTG
Mouse_Sfxn5_f	GGCTACTCCTACCTGTGCATA
Mouse_Sfxn5_r	ATCTGCGGGAAAAGGCTGATG
Mouse_18S_f	ATTAATCAAGAACGAAAGTCCCAGGT
Mouse_18S_r	TTTAAGTTTCAGCTTTGCAACCATACT
Primer for genotyping of SFXN2 Exon 4_f	ATTGATGGGGTCCTCTTGGG
Primer for genotyping of SFXN2 Exon 4_r	TACCTATAGTGTAGGGTTGTTGCG
Primer for genotyping of SFXN2 Exon 5_f	CAGGGAGGAACTTGGCATGT
Primer for genotyping of SFXN2 Exon 5_r	TCAGAAGCGCTGGAAGATGG
